# Heterogeneity of Baló’s concentric sclerosis: a study of eight cases with different therapeutic concepts

**DOI:** 10.1186/s12883-020-01971-2

**Published:** 2020-11-02

**Authors:** D. Tzanetakos, A. G. Vakrakou, J. S. Tzartos, G. Velonakis, M. E. Evangelopoulos, M. Anagnostouli, G. Koutsis, E. Dardiotis, E. Karavasilis, P. Toulas, L. Stefanis, C. Kilidireas

**Affiliations:** 1 Demyelinating Diseases Unit, 1st Department of Neurology, School of Medicine, Eginition Hospital, National and Kapodistrian University of Athens, Athens, Greece; 2 Research Unit of Radiology - 2nd Department of Radiology, National and Kapodistrian University of Athens, Athens, Greece; 3 Department of Neurology, University of Thessaly, University Hospital of Larissa, Larissa, Greece

**Keywords:** Baló’s concentric sclerosis, Multiple sclerosis, MRI, Immunotherapies

## Abstract

**Background:**

Baló’s Concentric Sclerosis (BCS) is a rare heterogeneous demyelinating disease with a variety of phenotypes on Magnetic Resonance Imaging (MRI). Existing literature lacks data especially on the therapeutic approach of the disease which we intended to elucidate by means of suggesting a new possible BCS classification and introducing different therapeutic concepts based on each BCS-subgroup characteristics.

**Methods:**

We present a retrospective study of eight treated patients with BCS-type lesions, emphasizing on MRI characteristics and differences on therapeutic maneuvers.

**Results:**

Data analysis showed: at disease onset the BCS-type lesion was tumefactive (size ≥2 cm) in 6 patients, with a mean size of 2.7 cm (± 0.80 SD); a coexistence of MS-like plaques on brain MRI was identified in 7 patients of our cohort. The mean age was 26.3 years (±7.3 SD) at disease onset and the mean follow-up period was 56.8 months (range 9–132 months). According to radiological characteristics and response to therapies, we further categorized them into 3 subgroups: a) Group-1; BCS with or without coexisting nonspecific white matter lesions; poor response to intravenous methylprednisolone (IVMP); treated with high doses of immunosuppressive agents (4 patients), b) Group-2; BCS with typical MS lesions; good response to IVMP; treated with MS-disease modifying therapies (2 patients), c) Group-3; BCS with typical MS lesions; poor response to IVMP; treated with rituximab (2 patients).

**Conclusions:**

Our study introduces a new insight regarding the categorization of BCS into three subgroups depending on radiological features at onset and during the course of the disease, in combination with the response to different immunotherapies. Immunosuppressive agents such as cyclophosphamide are usually effective in BCS. However, therapeutic alternatives like anti-CD20 monoclonal antibodies or more classical disease-modifying MS therapies can be considered when BCS has also mixed lesions similar to MS. Future studies with a larger sample size are necessary to further establish these findings, thus leading to better treatment algorithms and improved clinical outcomes.

**Supplementary Information:**

The online version contains supplementary material available at 10.1186/s12883-020-01971-2.

## Background

Baló’s concentric sclerosis (BCS) is a rare demyelinating disease, histopathologically characterized by large concentric lesions with circumferential rings of myelin loss alternating with rings of myelin preservation. BCS along with tumefactive Multiple Sclerosis (MS), Marburg and Schilder disease are considered types of fulminant demyelinating disorders of the central nervous system (CNS) [1]. BCS lesions have been considered to be a variant of pattern III of MS [2, 3]. Another hypothesis about BCS neuropathology is tissue preconditioning and hypoxia-like injury [4]. The hallmark of BCS on Magnetic Resonance Imaging (MRI) is alternating bands of different signal intensities on T2WI, alternating iso/hypointense and hyperintense signal, which resemble a “whirlpool” of concentric rings (“tree trunk” or “onion bulb” appearance) [5, 6]. Other typical radiological features include enhancement of the active layers of demyelination on contrast-enhanced T1-weighted images and diffusion restriction usually in the outermost rings on DWI [7]. These imaging findings are useful in the differential diagnosis of BCS from other tumefactive or atypical CNS lesions e.g. neoplasms, vasculitis [8]; advanced MRI techniques such as Magnetic Resonance Spectroscopy, Diffusion Tensor Imaging and Arterial Spin Labeling can also be applied in order to differentiate BCS from CNS neoplasms [9–12]. However, in addition to the typical BCS mass lesion on MRI, other MS-like plaques can also coexist [13]. We present a study of eight patients with a BCS-type lesion at onset, describing clinical, MRI and therapeutic parameters of the disease. Our analysis suggests distinct BCS subgroups with different responses to treatment.

## Methods

We conducted a retrospective study of eight treated patients with BCS that were diagnosed in the Demyelinating Diseases Unit at Eginition Hospital, Athens, Greece, from 2009 to the present. Diagnosis was based on clinical presentation, radiological features and exclusion of other disease mimics. All patients had a brain lesion with BCS-type characteristics (hyperintense-isointense-hypointense concentric rings on T2-weighted/FLAIR images) at symptom onset [5, 14]. The main measures considered were: 1) symptoms at onset, 2) size and gadolinium enhancement pattern of BCS-type lesion at onset and after treatment on MRI, 3) co-existence of MS-like plaques, 4) CSF oligoclonal band (OCB) presence, 5) clinical relapse, 6) long-term clinical response to treatment. Informed consent was obtained by all patients and approval had been obtained from the ethics committee of Eginition Hospital.

### Case series

#### Case 1

**Fig. 1:**
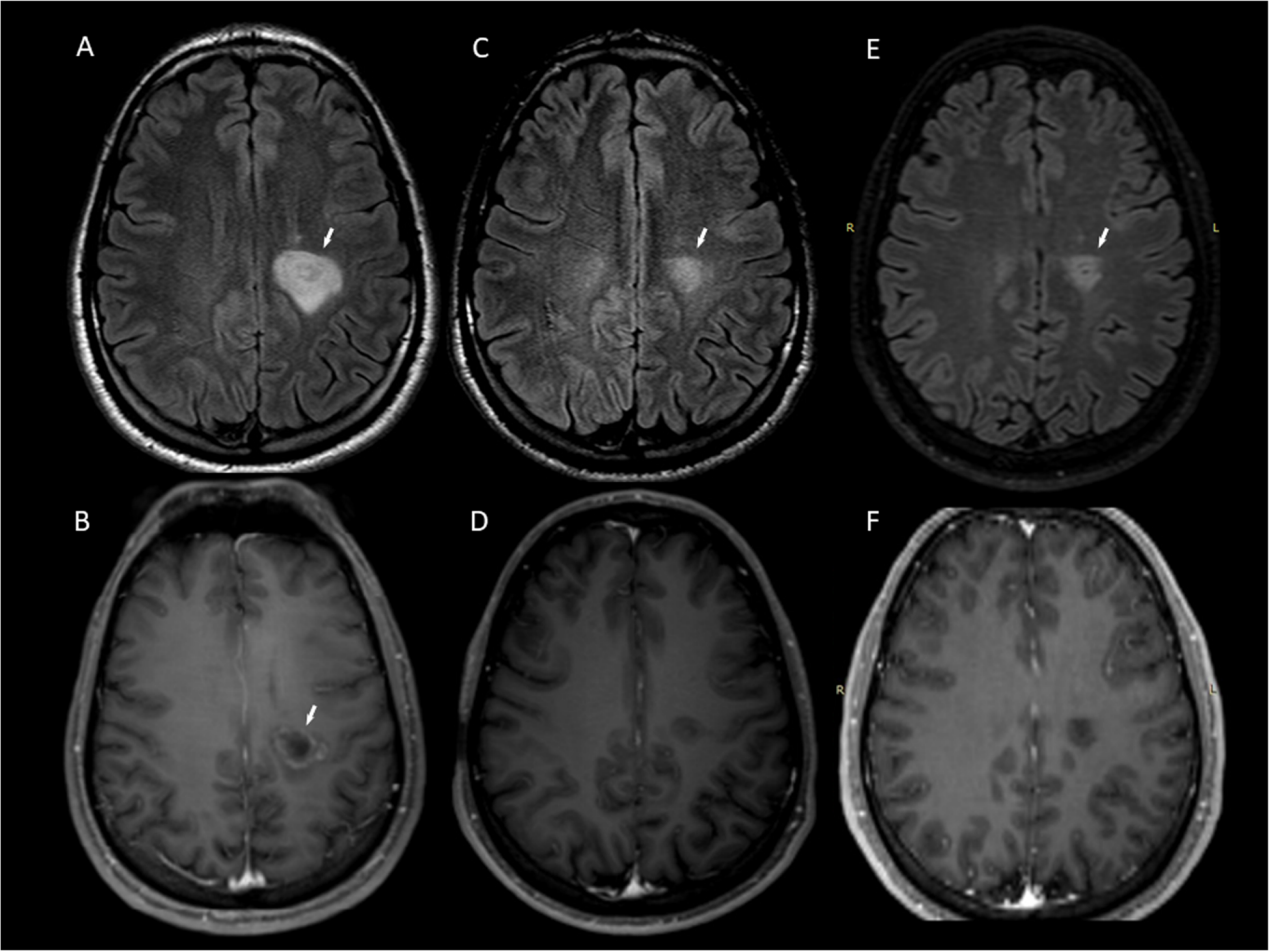
MRI of Case 1 showing BCS-type lesion before and after treatment with cyclophosphamide. Tumefactive BCS-type lesion of the left corona radiata at symptom onset (A) with partial Gd + of the outermost layer (B arrow). A BCS-type lesion with residual ill-defined concentric morphology (C arrow) and no Gd + (D) 15 months from onset, after 6 pulses of cyclophosphamide. BCS-type lesion 5 years from onset (E arrow, F). **a, c**: FLAIR images**. b, d**: T1-weighted contrast-enhanced images. **e**: 3D FLAIR image. **f**: 3D T1-weighted contrast-enhanced image. BCS: Baló’s concentric sclerosis, Gd+: gadolinium enhancement

A 24-year-old man presented with right-sided hemiparesis and hemianaesthesia; a tumefactive lesion (2.8 × 2.5 × 2.5 cm) with “onion bulb” appearance on FLAIR images and partial contrast enhancement (Gd+) of the outermost layer **(**Fig. 1a-b**)** of the left corona radiata and 3 periventricular non-enhancing (Gd-) lesions were detected on MRI. CSF analysis showed OCB presence. Intravenous methylprednisolone (IVMP) was administered with poor clinical response and subsequently, he underwent seven plasmapheresis sessions (PLEX) with MRI activity deterioration. After 6 cyclophosphamide pulses (800 mg/m^2^/pulse) a remarkable clinical outcome was observed; follow-up MRI (15 months from symptom onset) showed residual ill-defined concentric morphology of the BCS-type lesion (1.7 × 1.4 × 1 cm) and no Gd + **(**Fig. 1c-d**).** A significant decrease in size of the BCS lesion and 3 new T2-weighted hyperintense small lesions were observed during a 5-year-follow-up MRI **(**Fig. 1e-f**)** and the patient was started on glatiramer acetate over the last year **(**Table 1**)**.

**Table 1:** Synopsis of clinical, radiologic, laboratory and therapeutic characteristics in 8 patients with BCS

Case	Age at onset (years)	Sex	Symptoms at BCS onset	BCS-type lesion size (cm) at onset	Pattern of BCS-type lesion Gd+	Coexistence of brain MS-like plaques	Barkof criteria fulfillment at BCS onset	Spinal cord lesions at BCS onset	CSF OCBs	Time to follow-up from BCS onset (months)	Response to IVMP	Long-term Treatment
1	24	M	right-sided hemiparesis and hemianaesthesia	2.8	Peripheral Gd + at outer layer	(+)	No	(−)	(+)	85	poor	CYC
2	30	F	dysarthria,left facial palsy, left hand dexterity problems	3.6	Peripheral ring-like Gd + at outer layer	(−)	No	(−)	(+)	34	poor	CYC
3	41	M	Wernicke’s aphasia and right-sided hemianaesthesia	2.6	Peripheral ring-like Gd + at outer two layers	(+)	No	(−)	Not tested	132	poor	1st MTX 2nd GA
4	18	F	blurred vision	3.1	Peripheral incomplete ring-like Gd + at outer two layers	(+)	Yes	(−)	(+)	16	excellent	1st GA, 2nd NTZ
5	18	F	right-sided hemiparesis	2	Peripheral ringlike Gd + at outer layer	(+)	Yes	(+)	Not tested	127	good	1st IFN-beta-1b, 2nd NTZ
6	26	M	dysarthria	3.6	Peripheral incomplete ringlike Gd + at outer two layers	(+)	Yes	(−)	(−)	23	poor	RTX
7	26	F	gait instability, mild left hemiparesis	1.7	peripheral Gd + at outer layer	(+)	No	(+)	(+)	29	poor	RTX
8	27	M	left facial nerve palsy	1.8	partial ringlike Gd+	(+)	No	(−)	(−)	9	poor	CYC

Abbreviations: *BCS* Baló’s Concentric Sclerosis, *F* Female, M: Male, *Gd+* Gadolinium Enhancement, *CSF OCBs* Cerebrospinal Fluid Oligoclonal Bands, *(+)* presence, *(−)* absence, *MS* Multiple Sclerosis, *IVMP* intravenous methylprednisolone, *CYC* Cyclophosphamide, *MTX* Mitoxantrone, *RTX* Rituximab, *GA* Glatiramer acetate

#### Case 2

A 30-year-old woman, experienced dysarthria, left facial droop and left-hand dexterity problems for four days. MRI revealed a mass lesion (3.6 × 3.2 × 2.7 cm), with a concentric pattern, involving the right centrum semiovale with peripheral ring-like Gd + at outer layer **(Supplementary Figures** **1****A-C).** CSF studies demonstrated presence of OCBs. The patient was initially treated with 8 g of IVMP with poor response and subsequently with monthly IV pulses of cyclophosphamide (800 mg/m^2^/pulse) for 6 months with substantial clinical improvement. Three months after the last cyclophosphamide course the BCS-type lesion was significantly reduced in size (1.2 × 1.9 × 2 cm) **(Supplementary Figures** **1****D-F)** while the concentric MRI pattern almost disappeared; no further disease activity was observed on 25-month follow-up **(**Table 1**)**.

#### Case 3

A 41-year old male acutely developed Wernicke’s aphasia and right-sided hemianaesthesia due to a concentric lesion (2.6 × 1.8 × 1.2 cm), with an almost complete peripheral ring-like Gd + at outer two layers, involving the left corona radiata and the adjacent frontal subcortical white matter **(Supplementary Figures** **2****A-C)**. MS-like white matter lesions were also present (**Supplementary Figure** **2****D).** After 12 g of IVMP and subsequently four doses of IV mitoxantrone (10 mg/m^2^/3months) there was a good clinical and radiological response and he was subsequently switched to glatiramer acetate. Nine years later, brain MRI showed a significant decrease in size of the BCS-like lesion (1.9 × 1.6 × 1 cm) (**Supplementary Figures** **2****E-F)** and insignificant changes of the MS-like lesion load **(**Table 1**)**.

#### Case 4

**Fig. 2:**
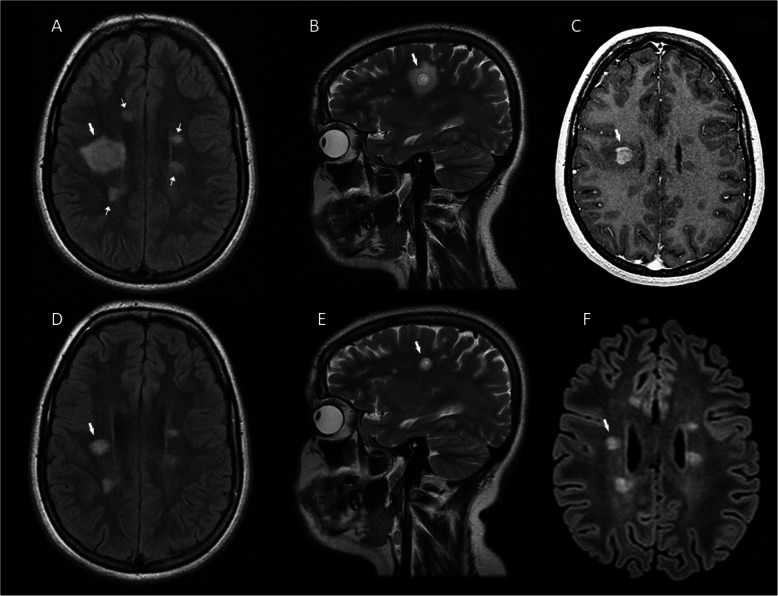
MRI of Case 4. Tumefactive BCS-type lesion in the right centrum semiovale (A and B thick arrow) with incomplete central solid Gd + along with incomplete ring-like Gd + of the outer two layers (C thick arrow) and multiple typical non-enhancing MS lesions supratentorially (A thins arrows). BCS-type lesion with effacement of the “onion bulb” pattern (D and E thick arrow) 2 months after BCS onset and after corticosteroid treatment. BCS-type lesion (F thick arrow) at the 7th month of natalizumab treatment and 13th from the onset. **a, d**: FLAIR images. **b, e**: T2-weighted images. **c**: T1-weighted contrast-enhanced images. **f**: 3D FLAIR image. BCS: Baló’s concentric sclerosis, Gd+: gadolinium enhancement

A 18-year-old female with a history of right retrobulbar optic neuritis and a second episode of left upper limb weakness with numbness and gait instability over the last year, presented with blurred vision. MRI revealed a tumefactive BCS-type lesion (3.1 × 2.5 × 1 cm), with incomplete central solid Gd + along with incomplete ring-like Gd + of the outer two layers in the right centrum semiovale. In addition, 10 smaller typical MS Gd- lesions were located supratentorially **(**Figs. 2a-c**)**. CSF OCBs were positive. The patient was treated with IVMP (9 g) with excellent clinical response. Follow-up MRI 2 months later demonstrated a significant reduction in size of the BCS lesion (1.5 × 8.4 × 1.1 cm) with no Gd + and effacement of the “onion bulb” pattern **(**Fig. 2d-e**)**. Glatiramer acetate was initiated and 3 months later she had a relapse with diplopia. Brain MRI demonstrated a small Gd + lesion, the patient received IVMP and treatment was switched to natalizumab; there was no MRI or clinical activity 11 months from natalizumab initiation (Fig. 2f) (Table 1).

#### Case 5

A 18-year-old woman presented with right-sided hemiparesis. MRI demonstrated a tumefactive lesion with “onion bulb” appearance (1.6 × 1.7 × 2 cm) with a peripheral complete ring-like Gd + in the left centrum semiovale, as well as multiple periventricular Gd- lesions (**Supplementary Figures** **3****A-C**). The patient was treated with IVMP with good clinical response and for the subsequent 8 years with IFN-beta-1b with no clinical relapse. IFN-beta-1b was discontinued due to pregnancy planning but 20-days postpartum she experienced left hemiparesis; on MRI the concentric pattern was less conspicuous (**Supplementary Figure** **3****D)** and 5 new Gd + brain lesions (larger lesion of 1.2 × 1.8 × 1.2 cm) were revealed (**Supplementary Figures** **3****E-F)**. She received IVMP with excellent clinical response and natalizumab was initiated with no disease activity during a 15-month follow-up **(Supplementary Figure** **3****G)** (Table 1).

#### Case 6

**Fig. 3:**
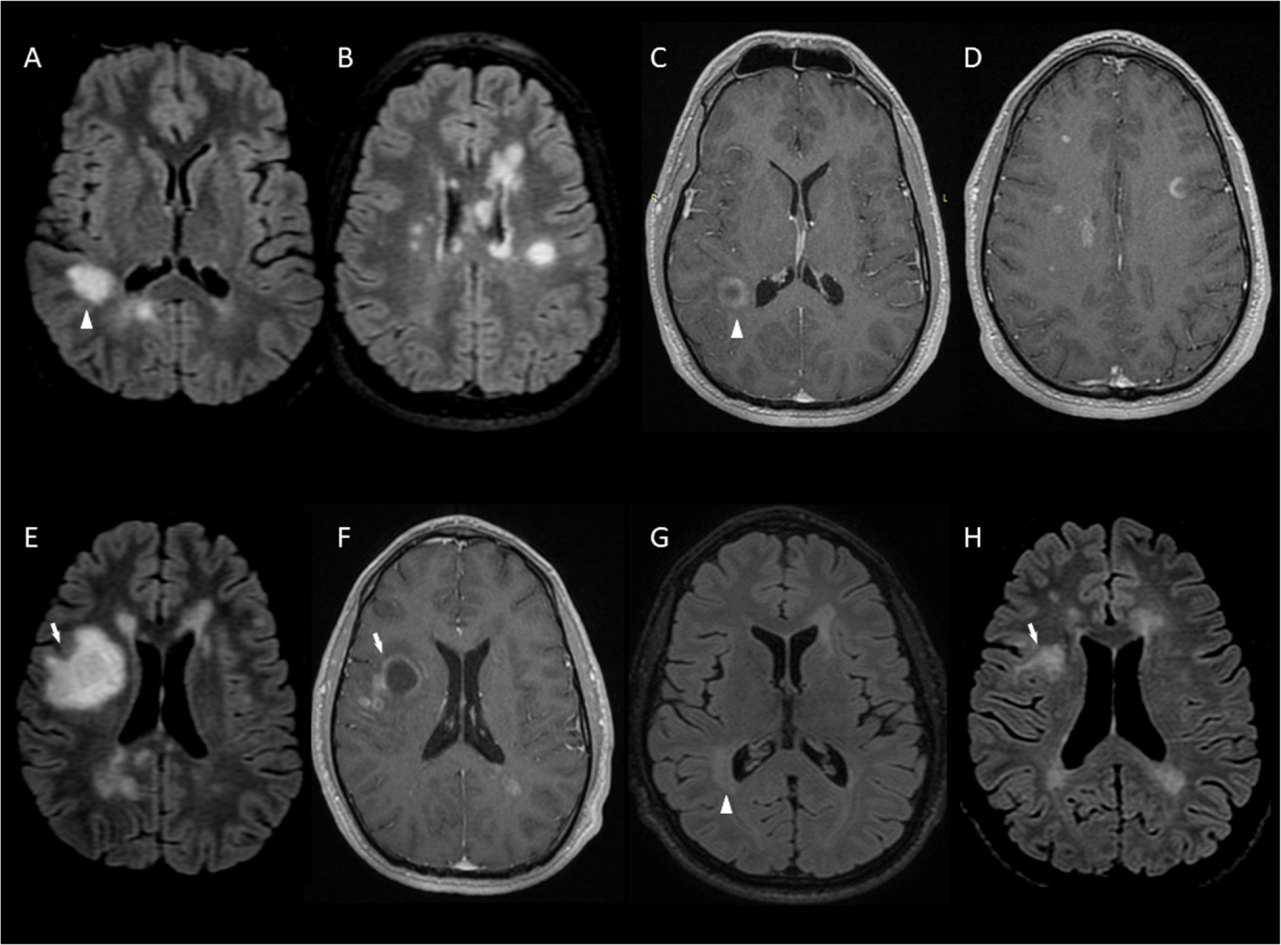
MRI of Case 6 showing coexistence of tumefactive and BCS-type lesions. At symptom onset a tumefactive lesion with incomplete ring Gd + in the right optic radiation-parietal lobe (A and C arrowhead) and multiple lesions (B); 14 with Gd + (D). A BCS-type lesion with incomplete ring Gd + (E and F thick arrow) in the right corona radiata evolved at a 2-months follow-up. BCS-type (H thick arrow) and tumefactive (G arrowhead) lesions at month 6 after rituximab initiation, one year from the first clinical episode. **a, b, e, g, h**: 3D FLAIR images. **c, d, f**: 3D T1-weighted contrast-enhanced images. BCS: Baló’s concentric sclerosis, Gd+: gadolinium enhancement

A 26-year old man presented with dysarthria for 12-h. On brain MRI a tumefactive lesion (2 × 2 × 2.3 cm) with incomplete ring Gd + in the right optic radiation-parietal lobe **(**Figs. 3a-d**)** were detected, along with 30 other supratentorial demyelinating lesions (14 showing Gd+). OCBs were absent and he was treated with IVMP. Follow-up MRI at 2 months demonstrated new brain Gd + lesions and a BCS-type lesion (3.2 × 3.6 × 2.8 cm) with incomplete ring Gd + in the right corona radiata **(**Fig. 3e-f**)**, whereas cervical spine MRI was normal. Due to new MRI activity, he received an additional course of IVMP and then 4-months from disease onset he was started on rituximab (375 mg/m^2^ IV once weekly × 4 doses) and subsequent doses (375 mg/m^2^ IV) once every 6 months. We noticed a significant decrease of both BCS-type and tumefactive lesion size on 6-months follow-up (Fig. 3g-h**)** and no disease activity over the next 16 months of rituximab treatment (Table 1).

#### Case 7

**Fig. 4:**
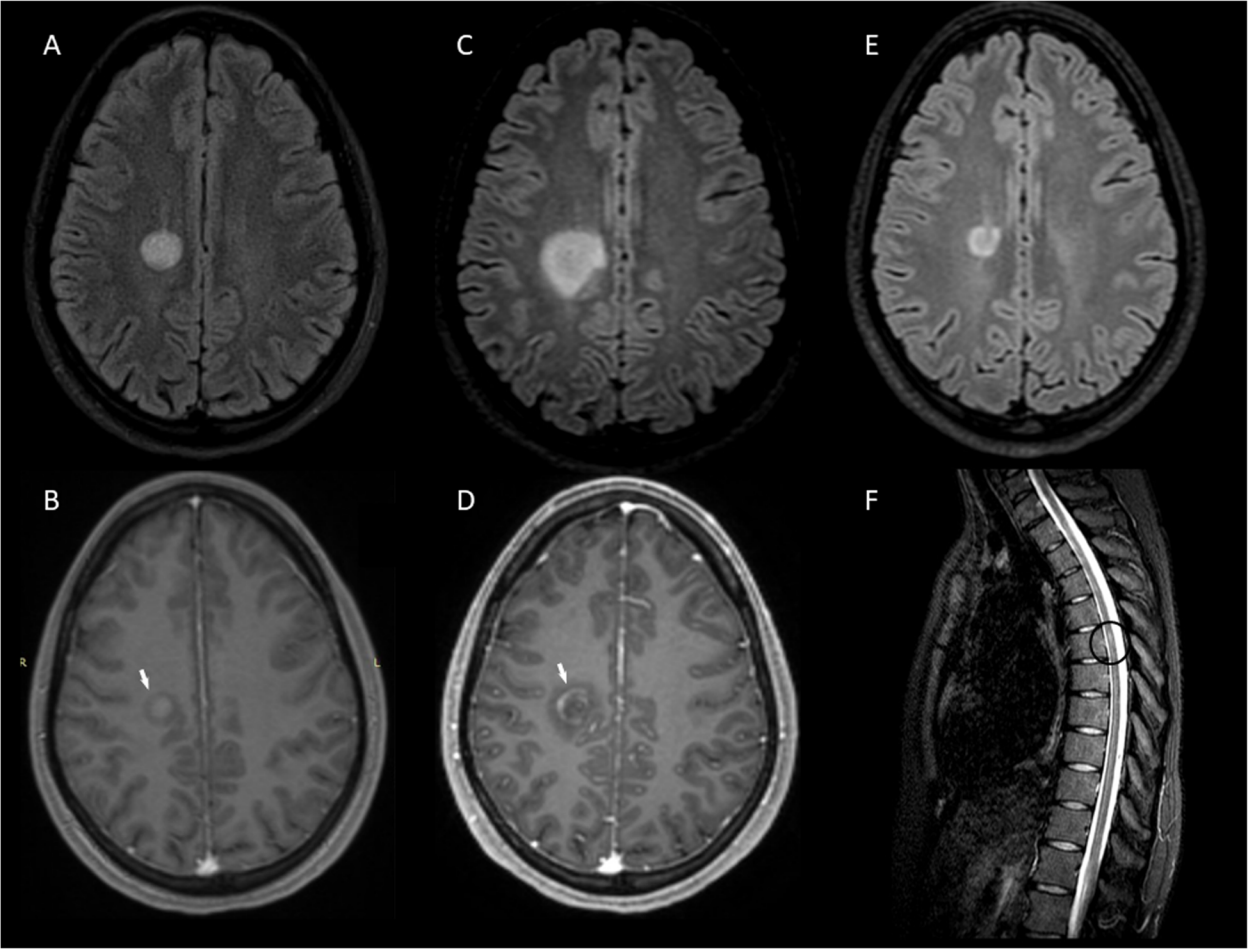
MRI of Case 7 before and after treatment with rituximab. A BCS-type lesion in the right centrum semiovale (A) with peripheral almost complete Gd + at outer layer (B arrow) at symptom onset. Follow-up MRI at 1 month, after corticosteroid treatment, showing enlargement of the BCS-type lesion (C) with Gd + (D arrow) and one lesion at Th6 level (F circle). BCS-type lesion (E) 4 months from the first rituximab course, 5 months from BCS onset. **a, c, e**: 3D FLAIR images. **b, d**: 3D T1-weighted contrast-enhanced images. **f**: STIR image. BCS: Baló’s concentric sclerosis, Gd+: gadolinium enhancement

A 26-year old woman developed acutely gait instability and mild left hemiparesis due to a BCS-type lesion (1.7 × 1.5 × 1.6 cm) in the right centrum semiovale with peripheral almost complete Gd + at outer layer (Fig. 4a-b). OCBs were positive and the patient was treated with IVMP with complete clinical recovery. One month later a significant increase in size of the concentric lesion (2.2 × 3.5 × 3.6 cm) was observed with ring-like Gd + at the outer two layers (Figs. 4c-d**)**, 2 new MS-like lesions and one spinal lesion at Th6 level were noted (Fig. 4f**)**. The patient was started on rituximab (375 mg/m^2^ IV once weekly × 4 doses) and 4-months later she was asymptomatic with a remarkable reduction in size of the BCS-type lesion (1.3 × 1.3 × 1.6 cm) (Fig. 4e). Rituximab infusions (375 mg/m^2^) once every 6 months achieved sustained disease control over the next 15 months (Table 1).

#### Case 8

A 27-year old man presented with left facial nerve palsy. Brain MRI revealed a BCS-like morphology lesion (1.8 × 1.1 × 1.6 cm) in the right centrum semiovale with partial ring-like Gd + and few Gd- lesions in cerebral hemispheres and right cerebellar peduncle (**Supplementary Figures** **4****A-D**). OCBs were absent and the patient was treated with IVMP (6 g) with clinical recovery in a few days. However, enlargement of the BCS-type lesion (2.1 × 1.3 × 1.6 cm) was noticed two months later **(Supplementary Figure** **4****E),** therefore a new IVMP course was administered (5 g). An insignificant decrease was observed in size after 4 months (**Supplementary Figures** **4****F-G)** and he was started on monthly cyclophosphamide infusions (800 mg/m2/pulse); there was no MRI activity or clinical relapse over a 3-month follow-up period under treatment (Table 1).

## Discussion and conclusions

The hallmark pathologic feature of BCS is the concentric rings of demyelination and myelin reservation, extending from a central lesion, usually around a venule [15]. It has been proposed that oxidative stress, hypoxia-induced tissue injury, chemotactic stimuli, astrocytopathy, loss of aquaporin-4, oligodendrocytopathy are potential causes of lesion formation [16]. Biopsies of BCS-type lesions have shown that the predominant immunopathologic feature is pattern-III demyelination (distal oligodendrocytopathy and apoptosis), and there is also preferential loss of MAG protein and high inducible nitric oxide synthase (iNOS) expression, by macrophages and microglia, as seen in ischemic stroke [2–4].

BCS has been considered to exhibit a monophasic severe course or typical Relapsing-Remitting MS (RRMS) course as well [17, 18]. It is disputable whether BCS is a distinct demyelinating disorder or a variant of MS or a subtype of tumefactive demyelination [19]. There are overlapping features as well as distinct pathological and radiological characteristics that suggest the existence of a spectrum among MS, tumefactive demyelination and BCS [20, 21]. It has been shown that BCS lesions or tumefactive lesions can arise during the clinical course of a typical RRMS [22].

Herein, we described 8 cases with BCS-type demyelinating lesions in the brain to emphasize the clinical heterogeneity and different therapeutic approaches of the disease. All patients (*n* = 8) presented at first clinical attack with a large lesion with alternating hyperintense-hypointense rings in T2WI/ FLAIR sequences; no more than two hyperintense-rings were observed. In 6 of them, the BCS-type lesion was tumefactive (size ≥2 cm), with a mean size of 2.7 cm (± 0.8 SD) with no mass effect. The assessment of BCS-type lesion topography showed that all were located in the cerebral white matter (WM), particularly in the corona radiata or centrum semiovale, extending to the adjacent subcortical WM but not to the cortex. In the majority of our cohort (87.5%, *N* = 7) we identified the coexistence of MS-like plaques on brain MRI with the BCS-type lesion at onset, while spinal lesions were detected in only 1 out of 7 BCS patients that were examined with spinal MRI. At BCS onset the mean age was 26.3 years(±7.3 SD) and clinical symptomatology was either severe e.g. Wernicke’s aphasia, hemiparesis, dysarthria or minor e.g. facial nerve palsy. In our study in 4 out of 6 patients, that underwent lumbar puncture, OCBs were detected. Mean follow-up after disease onset was 56.8 months (range 9–132 months).

Regarding therapeutic algorithms in our patient group in detail: cases 1, 2, 3 and 8 were treated with immunosuppression (Group-1; BCS with or without coexisting nonspecific white matter lesions; poor response to IVMP; treated with high doses of immunosuppresive agents). Case-2 had a single BCS-type lesion and case-1 and case-3 had a BCS-type lesion plus a few non-specific brain lesions (not fulfilling Barkhof criteria) and they showed a poor response to acute treatment with IVMP at disease onset. Interestingly, case-1 had MRI activity deterioration after plasma exchange (PLEX). Therefore, these 3 patients (cases 1–3) were treated with immunosuppression (with cyclophosphamide for cases 1 and 2, with mitoxantrone for case 3) with remarkable clinical response and significant reduction of BCS-type lesion size. Case-8 responded merely to IVMP, thus he was started on cyclophosphamide with MRI stability over a 3-month follow-up. Case-4 and case-5 (Group-2; BCS with typical MS lesions; good response to IVMP; treated with MS-disease modifying therapies) in addition to the BCS-type lesion had also typical MS lesions (fulfilling Barkhof criteria), they demonstrated a good outcome to IVMP at onset, following a RRMS clinical course and they were started on immunomodulatory treatment: glatiramer acetate and IFN-beta-1a respectively. Both patients were finally switched to natalizumab; case-4 due to a clinical relapse on month 3 and case-5 after 8 years of IFN-beta-1a and severe relapse at the post-partum period with a significant inflammatory component on brain MRI. Cases 6 and 7 (Group-3; BCS with typical MS lesions; poor response to IVMP; treated with rituximab) were treated with rituximab with a favorable response. Case-6 presented with tumefactive lesion along with typical MS lesions, responded poorly to steroids and shortly after steroid therapy a new tumefactive BCS-type lesion developed; the disease was controlled with rituximab therapy. Case-7 had a BCS-type lesion, with also MS-like lesions, which increased in size and became tumefactive after IVMP; she also responded well to rituximab. During the follow-up period no new BCS-type lesions were observed and all patients achieved sustained disease control with no clinical relapses.

Overall, in our cohort, tumefactive BCS-type lesions can be presented, with or without other white matter lesions, and these patients usually respond poorly to steroids but are highly responsive to cyclophosphamide (Group-1). One could speculate that the inflammatory microenvironment of MS is absent in these cases and there is a lack of cross-talk among cell-types dominating MS pattern-II and -III pathologies. These patients require high levels of immunosuppression with cyclophosphamide for better disease control. Taking into consideration the role of activated macrophages in the production of chemical mediators and cytokines that lead to demyelinating BCS lesions, cyclophosphamide is considered the only therapeutic strategy able to suppress this type of inflammation. Supportive of this notion is the observed disease deterioration after PLEX in one of our patients from Group-1. In this disease setting, type-II autoantibody dependent responses with IgG-deposition are not prominent and thus immediate autoantibody depletion cannot limit disease burden.

Our cohort also included patients who presented with BCS-type lesions along with typical MS lesions (Group-2) who responded well to steroids and subsequently were controlled under classical therapies for RRMS (glatiramer acetate, interferon-beta, natalizumab). This therapeutic result is highly indicative of the existence of a common pathophysiological mechanism underlying BCS-lesions and classical MS and supports the role of humoral responses in maintaining Balo milieu. Mixed pathologies with both patterns III and II have also been described in MS [2]. Triggering factors for the development of BCS-lesions are elusive, but one could speculate that there is a crosstalk in the inflammatory microenvironment that gives rise to the rings of demyelination and relative preservation of myelin.

Finally, we presented patients with BCS-type lesions along with typical MS lesions (Group-3) who were poor steroid responders, but eventually treated with successfully rituximab. These patients followed a clinical course and treatment-response closer to tumefactive MS. B-cell depletion therapy was effective in these patients implying a pivotal role of B cells not only in MS pathology but also in the orchestration of inflammatory changes seen in BCS lesions. B-cells apart from producing autoantibodies have a more complicated role in inflammatory response such as being antigen presenting cells and cytokine producing cells [23]. Until today, there is no clear pathogenetic role of B-cells contributing to the recurrent demyelinating bands.

Literature review reveals that there are only case reports for maintenance therapy after induction therapy with high doses of steroids in BCS. Drugs such as Interferon beta-1a, Mitoxantrone, Natalizumab and Fingolimod have been used,however the therapeutic benefit was more prominent when there were BCS-MS overlaps. Nevertheless, the follow up of these patients in clinical cases was narrow [24, 25]. Alemtuzumab treatment was associated with one death, further strengthening the hypothesis that the humoral responses are not prominent in BCS lesions, where innate immunity plays a central role with lipid-laden macrophages and giant multiple nuclei astrocytes predominating in BCS lesions [26]. The role of PLEX is controversial. There are reports of patients poorly treated with corticosteroids, in whom PLEX was a rational option because it is often considered as rescue therapy in more typical tumefactive lesions [24, 27]. Nevertheless, our patient deteriorated with PLEX. To the best of our knowledge, there are no data reporting the use of rituximab in BCS.

Our study introduces a new insight regarding the categorization of BCS into three subgroups depending on radiological features at onset and during the course of the disease in combination with the response to different immunotherapies. It is reasonable to consider MS disease-modifying therapies for BCS disease when a patient fulfills diagnostic criteria for RRMS. Initially, high doses of corticosteroids are recommended in these patients for disease remission. For patients who respond poorly to steroids, rituximab is effective. On the other hand, in those patients with solitary masses without evidence of typical MS lesions instigates the need for immediate immunosuppression with cyclophosphamide after corticosteroids. PLEX in these patients is not suggested due to its failure to control the disease. Finally, we speculate that BCS is a heterogeneous disease of which one subgroup shares common features with MS (OCBs, MS-type lesions, relapses, humoral responses) while another subgroup is characterized by a different etiopathogenesis (activated macrophages and astrocytes). Since we analysed a small patient-group of this rare disease, definite conclusions regarding BCS classification in distinct subgroups cannot be drawn with safety, so future studies with a larger sample size are necessary to further establish these findings, thus leading to better treatment algorithms and improved clinical outcomes.

## Supplementary Information

**Additional file 1:** **Supplementary Figure 1.** MRI of Case 2 showing BCS-type lesion before and after treatment with cyclophosphamide. **Supplementary Figure 2.** MRI of Case 3 showing BCS-type lesion before and after mitoxantrone treatment. **Supplementary Figure 3.** MRI of Case 5. **Supplementary Figure 4.** MRI of Case 8.

## Data Availability

Data can be available upon request by the corresponding author.

## References

[1] Razek A, Elsebaie NA (2020). Imaging of fulminant demyelinating disorders of the central nervous system. J Comput Assist Tomogr.

[2] Lucchinetti C, Bruck W, Parisi J, Scheithauer B, Rodriguez M, Lassmann H (2000). Heterogeneity of multiple sclerosis lesions: implications for the pathogenesis of demyelination. Ann Neurol.

[3] Popescu BF, Lucchinetti CF (2012). Pathology of demyelinating diseases. Annu Rev Pathol.

[4] Stadelmann C, Ludwin S, Tabira T, Guseo A, Lucchinetti CF, Leel-Ossy L (2005). Tissue preconditioning may explain concentric lesions in Balo's type of multiple sclerosis. Brain.

[5] Caracciolo JT, Murtagh RD, Rojiani AM, Murtagh FR (2001). Pathognomonic MR imaging findings in Balo concentric sclerosis. AJNR Am J Neuroradiol.

[6] Sarbu N, Shih RY, Jones RV, Horkayne-Szakaly I, Oleaga L, Smirniotopoulos JG (2016). White matter diseases with radiologic-pathologic correlation. Radiographics.

[7] Kavanagh EC, Heran MK, Fenton DM, Lapointe JS, Nugent RA, Graeb DA (2006). Diffusion-weighted imaging findings in Balo concentric sclerosis. Br J Radiol.

[8] Abdel Razek AA, Alvarez H, Bagg S, Refaat S, Castillo M (2014). Imaging spectrum of CNS vasculitis. Radiographics.

[9] Abdel Razek AAK, Talaat M, El-Serougy L, Gaballa G, Abdelsalam M (2019). Clinical applications of arterial spin labeling in brain tumors. J Comput Assist Tomogr.

[10] Abdel Razek AAK, Talaat M, El-Serougy L, Abdelsalam M, Gaballa G (2019). Differentiating Glioblastomas from solitary brain metastases using arterial spin labeling perfusion- and diffusion tensor imaging-derived metrics. World Neurosurg.

[11] Abdel Razek AAK, El-Serougy L, Abdelsalam M, Gaballa G, Talaat M (2019). Differentiation of primary central nervous system lymphoma from Glioblastoma: quantitative analysis using arterial spin labeling and diffusion tensor imaging. World Neurosurg.

[12] Yeo CJJ, Hutton GJ, Fung SH (2018). Advanced neuroimaging in Balo's concentric sclerosis: MRI, MRS, DTI, and ASL perfusion imaging over 1 year. Radiology case reports.

[13] Wallner-Blazek M, Rovira A, Fillipp M, Rocca MA, Miller DH, Schmierer K (2013). Atypical idiopathic inflammatory demyelinating lesions: prognostic implications and relation to multiple sclerosis. J Neurol.

[14] Karaarslan E, Altintas A, Senol U, Yeni N, Dincer A, Bayindir C (2001). Balo's concentric sclerosis: clinical and radiologic features of five cases. AJNR Am J Neuroradiol.

[15] Barz H, Barz U, Schreiber A (2016). Morphogenesis of the demyelinating lesions in Balo's concentric sclerosis. Med Hypotheses.

[16] Takai Y, Misu T, Nishiyama S, Ono H, Kuroda H, Nakashima I (2016). Hypoxia-like tissue injury and glial response contribute to Balo concentric lesion development. Neurology..

[17] Hardy TA, Reddel SW, Barnett MH, Palace J, Lucchinetti CF, Weinshenker BG (2016). Atypical inflammatory demyelinating syndromes of the CNS. Lancet Neurol.

[18] Ayrignac X, Letourneau-Guillon L, Carra-Dallière C, Duquette P, Girard M, Poirier J (2020). From Baló's concentric sclerosis to multiple sclerosis: a series of 6 patients. Multiple Sclerosis Relat Disord.

[19] Jarius S, Wurthwein C, Behrens JR, Wanner J, Haas J, Paul F (2018). Balo’s concentric sclerosis is immunologically distinct from multiple sclerosis: results from retrospective analysis of almost 150 lumbar punctures. J Neuroinflam.

[20] Cai X, Xu J, Xu J, Pan D (2015). Serial magnetic resonance imaging representation in a Balo's concentric sclerosis. J Neurol Sci.

[21] Hardy TA, Tobin WO, Lucchinetti CF (2016). Exploring the overlap between multiple sclerosis, tumefactive demyelination and Balo's concentric sclerosis. Multiple Sclerosis.

[22] Barnett MH, Prineas JW (2004). Relapsing and remitting multiple sclerosis: pathology of the newly forming lesion. Ann Neurol.

[23] Baker D, Pryce G, Amor S, Giovannoni G, Schmierer K (2018). Learning from other autoimmunities to understand targeting of B cells to control multiple sclerosis. Brain.

[24] Hardy TA, Beadnall HN, Sutton IJ, Mohamed A, Jonker BP, Buckland ME (2015). Balo's concentric sclerosis and tumefactive demyelination: a shared immunopathogenesis?. J Neurol Sci.

[25] Capello E, Mancardi GL (2004). Marburg type and Balo's concentric sclerosis: rare and acute variants of multiple sclerosis. Neurol Sci.

[26] Brown JW, Coles AJ, Jones JL (2013). First use of alemtuzumab in Balo's concentric sclerosis: a case report. Multiple Sclerosis.

[27] Sekijima Y, Tokuda T, Hashimoto T, Koh CS, Shoji S, Yanagisawa N (1997). Serial magnetic resonance imaging (MRI) study of a patient with Balo's concentric sclerosis treated with immunoadsorption plasmapheresis. Multiple Sclerosis.

