# Supplementary material for: Heterogeneity of Baló’s concentric sclerosis: a study of eight cases with different therapeutic concepts
Source: BMC Neurol. 2020 Nov 2;20:400. doi: 10.1186/s12883-020-01971-2 (PMC7604966; doi:10.1186/s12883-020-01971-2)
Supplement: Supplementary file 1 — Additional file 1: Supplementary Figure 1. MRI of Case 2 showing BCS-type lesion before and after treatment with cyclophosphamide. Supplementary Figure 2. MRI of Case 3 showing BCS-type lesion before and after mitoxantrone treatment. Supplementary Figure 3. MRI of Case 5. Supplementary Figure 4. MRI of Case 8. [file 12883_2020_1971_MOESM1_ESM.docx]

**Additional Data:**

**Supplementary Figures**


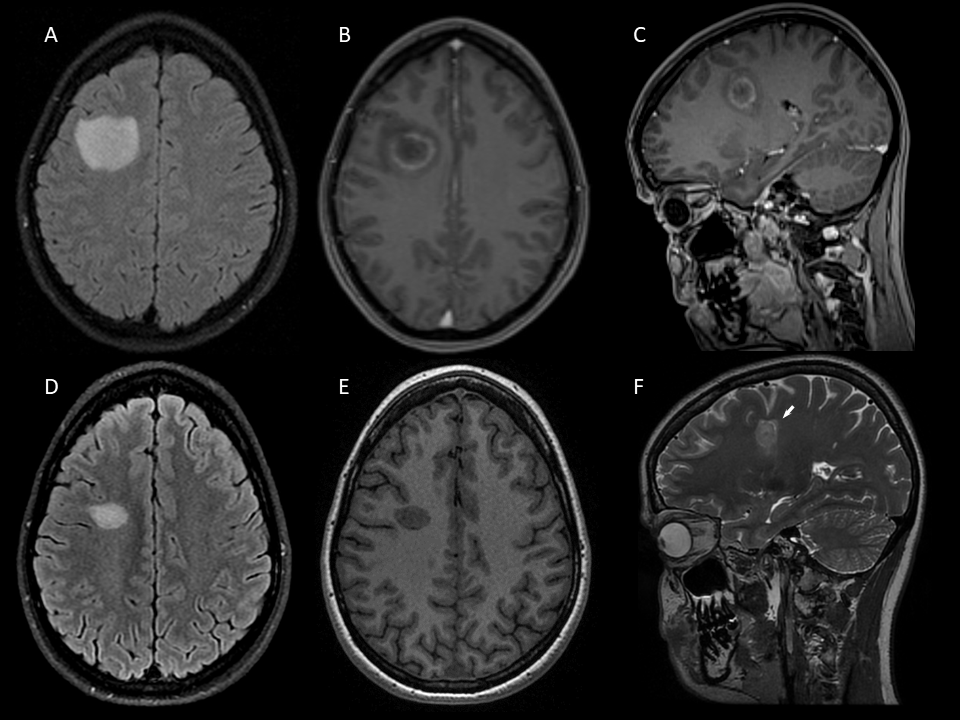


**Supplementary Figure 1: MRI of Case 2 showing BCS-type lesion before and after treatment with cyclophosphamide**

Tumefactive BCS-type lesion in the right centrum semiovale at symptom onset (Α) with peripheral ring-like Gd+ at outer layer (B, C). BCS-type lesion (D, F arrow) with no Gd+ (E) 10 months from onset and 3 months after the 6th cyclophosphamide course.

A: FLAIR image. B, C, E: T1-weighted contrast-enhanced images. D: 3D FLAIR image. F: T2-weighted image.

BCS: Baló’s concentric sclerosis, Gd+: gadolinium enhancement.


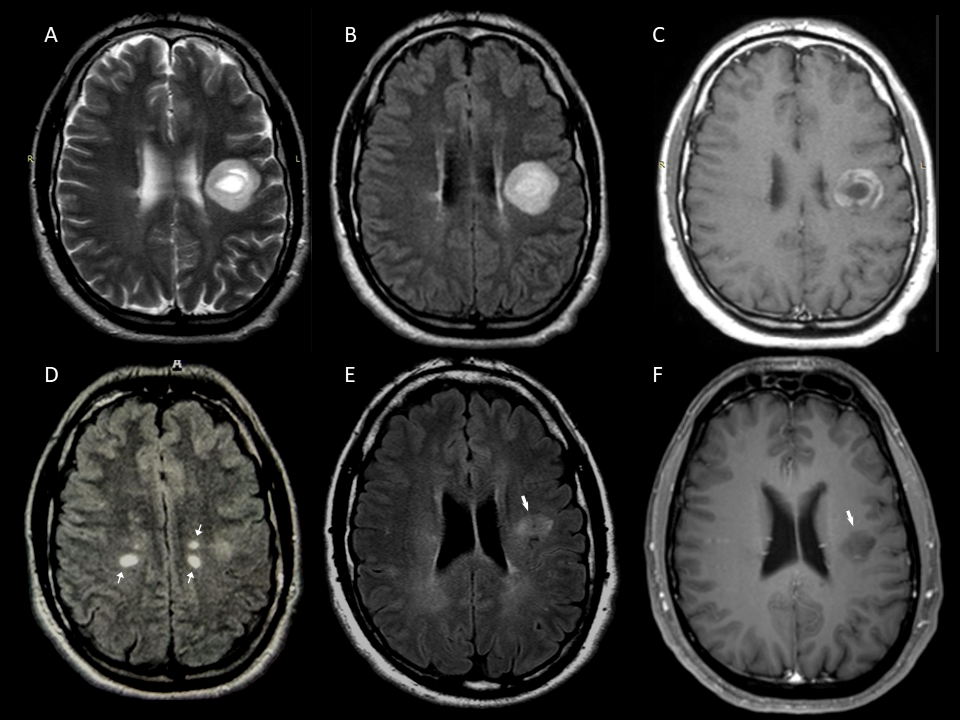


**Supplementary Figure 2: MRI of Case 3 showing BCS-type lesion before and after mitoxantrone treatment**

A concentric tumefactive lesion involving the left corona radiata and the adjacent frontal subcortical white matter (A, B) with an almost complete peripheral ring-like enhancement at outer two layers (C) and presence of MS-like white matter lesions (D thin arrows) at symptom onset. Nine years from disease onset (E thick arrow, F thick arrow), after mitoxantrone treatment (4 pulses) during the first year.

A: T2-weighted image. B, D, E: FLAIR images. C: T1-weighted contrast-enhanced image.

F: 3D T1-weighted contrast-enhanced image.

BCS: Baló’s concentric sclerosis, Gd+: gadolinium enhancement.


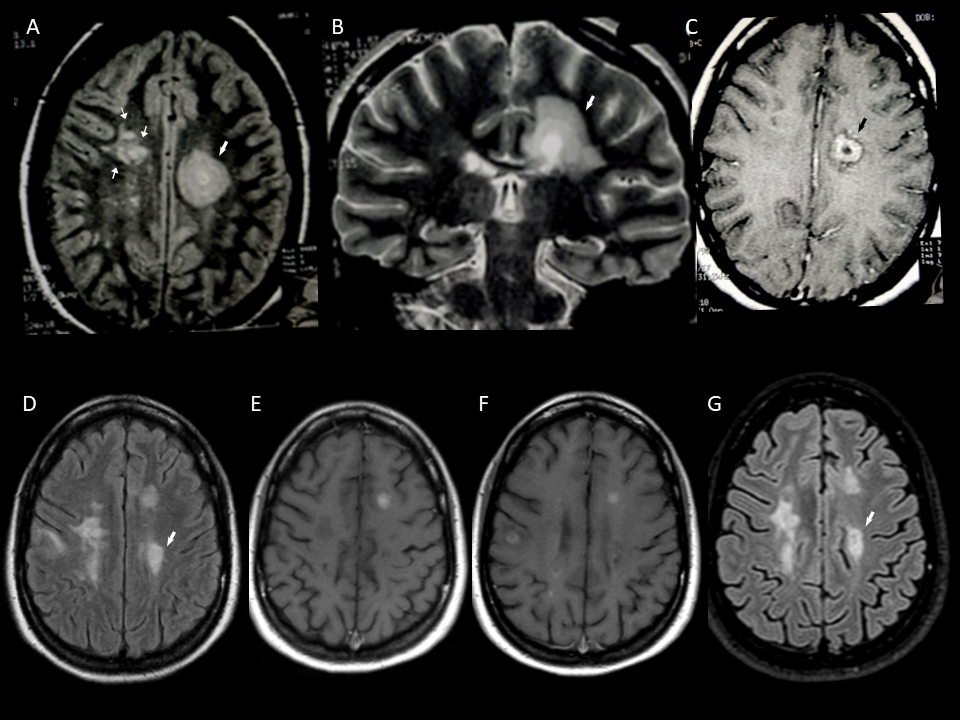


**Supplementary Figure 3: MRI of Case 5**

BCS-type at the onset with typical “onion bulb’’ appearance (A and B thick arrow) in the left centrum semiovale, with Gd+ (C thick arrow) and multiple periventricular lesions (A thin arrows). Nine years after BCS onset and 20 days postpartum the patient had a clinical relapse; the BCS-type lesion (D thick arrow) and 5 new Gd+ brain lesions (E, F). BCS-type lesion (G thick arrow) at month-11 of natalizumab treatment and 10 years from disease onset.

Α, D, G: FLAIR images. Β: T2-weighted image. C, E, F: T1-weighted contrast-enhanced images.

G: 3D FLAIR image.

BCS: Baló’s concentric sclerosis, Gd+: gadolinium enhancement.

**
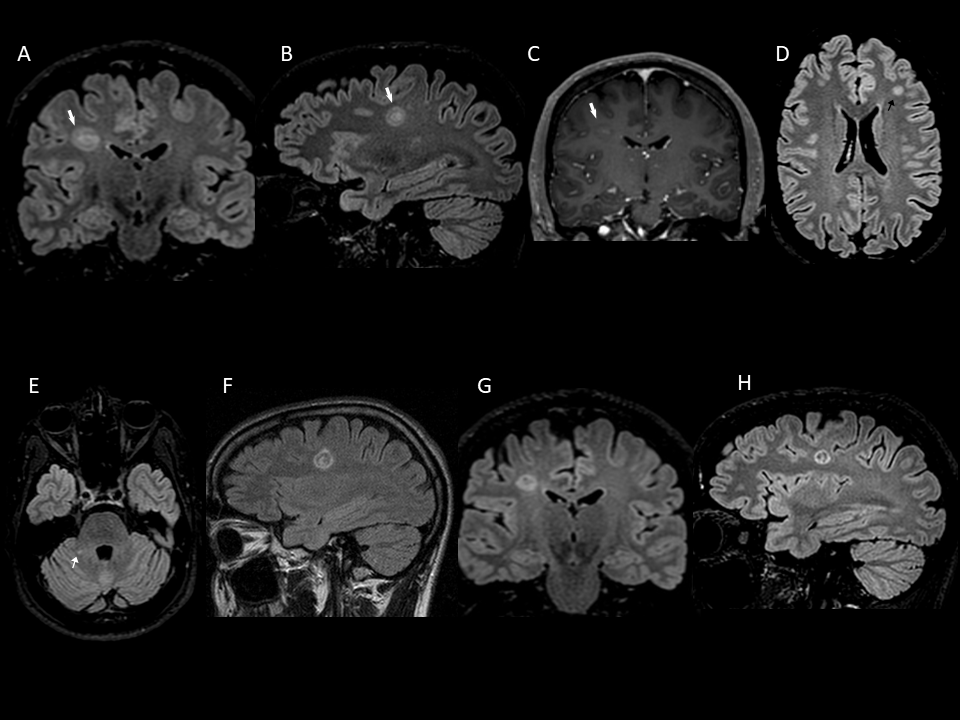
**

**Supplementary Figure 4: MRI of Case 8**

BCS-type lesion in the right centrum semiovale (A and B thick arrow) with partial ring-like Gd+ (C thick arrow) and few lesions with no Gd+ in cerebral hemispheres and right cerebellar peduncle (D black arrow and E thin arrow). Enlargement of the BCS lesion at 2-month follow-up (F) after intravenous corticosteroids. The BCS-type lesion after 4 months following a second corticosteroid course (G, H).

Α, D, E, F, G, H: 3D FLAIR images. C: 3D T1-weighted contrast-enhanced image.

BCS: Baló’s concentric sclerosis, Gd+: gadolinium enhancement.
